# Anti-MRSA activity of a bioactive compound produced by a marine *Streptomyces* and its optimization using statistical experimental design

**DOI:** 10.22038/ijbms.2019.33880.8058

**Published:** 2019-09

**Authors:** Hamed Norouzi, Mohammad Rabbani Khorasgani, Abolghasem Danesh

**Affiliations:** 1 Department of Biology, University of Isfahan, Isfahan, Iran; 2 Biotechnology Research Center, Pharmaceutical Technology Institute, Mashhad University of Medical Sciences, Mashhad, Iran

**Keywords:** Experimental designs, Marine microbiology, Methicillin-resistant - Staphylococcus aureus, Natural products Fermentation

## Abstract

**Objective(s):**

To address the alarming problem of methicillin-resistant *Staphylococcus aureus* (MRSA), herein, a marine *Streptomyces* capable of producing an anti-MRSA compound has been studied.

**Materials and Methods:**

Strain MN41 was morphologically and physiologically characterized and then, molecularly identified using 16SrRNA analysis. To produce the bioactive compound in large scale, a kind of submerged liquid fermentation was adopted. The antibacterial agent was purified using a silica gel column followed by a semi-preparative HPLC and the isolated metabolite was identified using mass spectrometry, Nuclear magnetic resonance (NMR) and Fourier-transform infrared (FTIR). Finally, the production process was subjected to a two steps optimization using Plackett-Burman design (PBD) and Response Surface Method (RSM), respectively. In addition, the antitumor activity of the active agent was studied.

**Results:**

The purified compound with a molecular weight of 421.2 was identified as a natural pyrrole-derivative. The optimization revealed a significant effect for starch, pH, calcium carbonate and peptone on the production of this anti-MRSA compound and resulted in a 218% increase in the production yield.

**Conclusion:**

The isolated pyrrole-derivative showed a remarkable activity against MRSA and also showed some promising anti-tumor activity.

## Introduction

Multi-drug resistant pathogens have been a serious problem in the health care systems. The aberrant usage of antibiotics is a major reason for the increasing number of antimicrobial-resistant pathogens. Therefore finding effective alternatives such as novel drug against multi-drug resistant bacteria is a must (1). Among pathogens that developed resistance toward antimicrobials, *Staphylococcus aureus* is an important one, which is responsible for different infectious disease including sore throat, osteomyelitis, pimples, endocarditis, and bacteremia (2), and the methicillin-resistant *S. aureus* (MRSA) strains, which was first discovered in Britain in 1961 and then found widespread in hospitals all around the world, has been one of the most serious drug-resistant pathogens, globally (3). 

Marine Actinomycetes are a source of novel chemical structures (4) with a diverse range of biological activities, e.g. antibacterial, anticancer, and immunosuppressive (5), and *Streptomyces*, as the largest genus of Actinomycetes, are a well-known source for such purpose (6-9). In spite of insignificant screening studies, the discovery speed of active biomolecules produced by marine *Streptomyces* has outmatched that of isolated from other terrestrial actinomycets, (10). Among them,* Streptomyces *Sp. MN41 produces active agents with antibacterial activity against both Gram-positive and Gram-negative microorganisms (11). 

An important aspect in production of active compound by microorganisms is that the species (or even the strain) as well as the cultivation conditions can significantly affect the final yield (12, 13), i.e. small changes in media composition may influence both the quality and the quantity of secondary metabolites production and general active compound profile of a certain microorganism (14, 15). Therefore, after screening/selection of a proper candidate microorganism, optimizing the growth and production conditions is an essential step for improving the production of antimicrobial compounds. This is normally carried out using one factor at a time method. OFAT is only applicable when the number of variables which influence the production yield are few, but it is inefficient if many factors should be considered (then, a large number of trails is required)(16). In addition, it is not a comprehensive method for describing the combined effect of thos evariables (15). Biostatistical methods such as Plackett-Burman design and Response Surface method can conquer these drawbacks. Plackett-Burman design (PBD) is a useful technique to identify the significant factors involved in the process while RSM is a potent method to determine the optimal levels of those variables and figure out the interactions between them (15, 17). In the present study, the isolation, structure elucidation, and biological activity of the bioactive compound(s) produced by a marine *Streptomyces *sp. MN41 is investigated. The effect of critical components on the production was evaluated using PBD in order to determine the significant ones. Afterward, the most significant influencing factors were optimized by RSM using a central composite design (CCD).

## Materials and Methods

***Microorganism***

The actinomycete strain used in this work, *Streptomyces *sp. MN41, was isolated from Caspian Sea sediment using starch casein agar (SCA) medium (11). The strain MN41 was kept alive on SCA agar slants incubated at 4 ^°^C. Methicillin-resistant *Staphylococcus aureus *subsp.* aureus *(ATCC^®^ 33591™) (www.atcc.org) was used as indicator strain.

***Strain characterization***

Cultural characteristics of the strain MN41 were determined according to the standard methods. Growth characteristics were ascertained on various International *Streptomyces* Project media (ISP media), including malt extract agar (ISP2), oat meal agar (ISP3), inorganic salts starch agar (ISP4), glycerol asparagine agar (ISP5), Peptone yeast extract agar (ISP6) and tyrosine agar (ISP7), after incubation at 28±2 ºC for 10–15 days (18). 

***16S rRNA analysis***

The molecular identification of MN41 was performed by extracting the genomic DNA with a standard bead beating method (19). The quality and quantity of the extracted DNA were determined by spectrophotometer and separated by electrophoresis run on a 0.8% (w/v) agarose gels. Next, the 16S rDNA gene was PCR amplified using universal bacterial 16SrDNA primers: PA5′-GAGTTTGATCCTGGCTCAG-3′ and PH 5′-AGGAGGTGATCCAGCCGCA- 3′. The thermocycler program was: starting from denaturation step at 95 ^°^C for the first 5, followed by 35 cycles, of 1 min at 95 ^°^C, 1 min at 50 ^°^C and at 2 min at 72 ^°^C, and finished by the final extension step at 72 ^°^C for 5 min. A GeneJet PCR purification kit (Thermo Scientific, Lithuania) was used to purify the PCR products according to the manufacturer’s instructions and then was sequenced by GATC Biotech (Germany). The National Center for Biotechnology Information (NCBI) was used for retrieving reference sequences, and the degree of genetic similarity was assessed using the BLAST program (www.ncbi.nlm.nih.gov/blst). Afterward, ClustalX, as a multiple sequence alignment program, was used for genetic alignment. Finally, the phylogenetic tree was constructed using MEGA5 (20).

***Production and purification of the bioactive compound***

The strain was grown under submerged liquid fermentation using modified A1BFe+C medium (starch 10, yeast extract 4, peptone 2, KBr 0.1, Fe_2 _(SO_4_)_3_·4H_2_O 0.04, CaCO_3_ 1, sea salt 30 (g L^−1^) and pH adjusted to 7.0). A single colony of *Streptomyces *sp. MN41 was inoculated in a 250 ml Erlenmeyer flask containing 50 ml of the seed medium followed by incubation at 28 ^°^C for 54 hr on a shaker with a speed of 180 rpm and then used as a seed stock for antibacterial compound production. Six ml of the seed culture (6%) were transferred into 500 ml Erlenmeyer flask containing 94 ml of the production medium (the same as the seed medium) in, subsequently placed in a rotary shaker incubator (180 rpm) at 28 ^°^C for 85 hr. The fermentation broth from all flasks (total volume = 5l) was then collected and centrifuged (Sigma) at 16000×g for 10 min. The collected supernatant was extracted three times with the same volume of ethyl acetate, while the pelleted mycelia cake was extracted three times with methanol (2 l). Then, the organic solvent was removed and the samples (fermentation broth and mycelia cake) were combined again and considered as the crude extracts (7 g). The ethyl acetate crude extract was reconstituted in 10 ml and then was subjected to a normal-phase Si-gel open CC column (6250 mesh, 35 g). The elution was performed stepwise using 2 bed-volume of methanol/ethyl acetate mixture with different ratio of methanol: ethyl acetate including 0:1; 1:9; 2:8; 1:3; 1:2, 1:1 and 1:0 v/v. Finally, all fractions were concentrated by evaporating the organic solvent and their antibacterial activity was tested against MRSA (21).

***Identification analysis***

*Thin layer chromatography (TLC)*

TLC was used for screening and selection of the best pure active fraction. It was performed on a silica gel 60 F_254_ plate (Merck), where one microliter of each concentrated-active fraction was applied on the sheet and developed using chloroform: methanol (8:1) as the solvent system, and the chromatogram was visualized in a UV chamber (254 nm) (22). Ninhydrin was used to detect peptides or protein structures, if any was present. Retention factor (Rf) of the compound was also determined.

*HPLC analysis*

The best active fractions that showed only one band in TLC analysis were first analyzed using semi-preparative HPLC (KNAUER) equipped with an ACE Semi-Preparative HPLC C18 column, (25 cm × 4.6 mm I.D, 7.75 µm) equipped with a UV-detector at 254 nm, with methanol as solvent and a flow rate of 9 ml/min for a period of 20 min. The water/methanol mix was at a starting point of 20% and the gradient was proceed to 30% methanol at the end of first 5 min and then allowed to reach 100% methanol during the next 5 min. The elution was continued with 100% methanol during the third 5 min and finally, the gradient attenuated to 20% methanol during the last 5 min followed by 2 min equilibration period. Eight sub-fractions were obtained and analyzed on TLC and tested for antibacterial activity (23).

*Mass spectrometry*

Five µl of each HPLC fractions was injected in a mass spectrometer (QTrap 3200, AB SCIEX) equipped with a TurboSpray (TIX), with methanol containing 0.1% trifluoroacetic acid as the carrier solvent, and was used for determining the molecular mass of the bioactive compound and obtaining structural information from the device. The ion source was set to positive ion mode followed by adjusting the quadrupole system to scan between m/z 50-1700 in Q-MS mode. The m/z value of selected precursor ion was fragmented under argon pressure (i.e. collision induced dissociation) and scanned in a range of m/z 50-1000 as a production mode (24). 

*NMR analysis*

^1^H, ^13^C, and 2D NMR spectroscopic data were obtained on a BRUKER 300MHz AVANCE III spectrometer in a methanol-d4 solvent. Offline processing was conducted using topspin NMR software by Bruker BioSpin 2011 (iNMR, http://www.inmr.net).

*FTIR determination*

KBR pellet method was used to take an IR spectrum of the compound in the range of 4000-400 cm^-1^ by PerkinElmer spectrometer.

*Minimum inhibitory concentration (MIC) and minimum bactericidal concentration (MBC)*

A 24 hr culture of MRSA was prepared using nutrient broth and its turbidity was adjusted to 10^6^ CFU/ml by sterile normal saline 0.9%. Different concentrations of the purified compound (5-0.039 mg/ml) were prepared by diluting in Mueller-Hinton broth (MHB, Merck). At first, 180 μl of each concentration was poured in a 96-well plate, then 20 μl of the adjusted bacterial suspension (10^6^ CFU/ml) was added and incubated at 37 ^°^C. Wells containing only medium were used as negative controls while MRSA suspension mixed to the MHB was used as a positive control. After 24 hr, 20 μl of the colorimetric indicator 2,3,5-triphenyl tetrazolium chloride 5 mg/ml (Merck) was added to the bacterial growth and the plates were incubated for 1 hr at 37 ^°^C. The MIC was defined as the minimum concentration of active compound that resulted in no color-changing in the medium. Finally, 20 μl of the suspensions from no-color-changed wells was inoculated on Mueller-Hinton agar plates to determine the MBC (25).

*Cytotoxicity assay*

MTT assay was used to evaluate the effect of the pure active compound on the proliferation of the cancer cell lines performed. Vero and Hela cell lines obtained from Razi Vaccine and Serum Research Institute (Iran) were cultured in DMEM medium. Cell culture media were supplemented with 10% calf serum and incubated at 37 ^°^C in 5% CO_2_ incubator. With the aim of having 80-90% confluence after 48 hr treatment, optimum numbers of the cell lines were seeded in each well according to their growth rate. After 12 hr, the old media were replaced by 100 µl of fresh media containing different concentrations of the extract (3.9, 7.81, 15.62, 31.25, 62.5, 125, 250, 500 and 1000 µg/ml). The plates were then incubated in a 5% CO_2_ incubator at 37 ^°^C for 72 hr. Tetrazolium bromide solution (Sigma, USA) was then added at 0.5 mg/ml final concentration and the mixture was incubated in the dark for 4 hr. The media were then aspirated from each well and 100 µl DMSO (Merck, Darmstadt, Germany) was added to solve formazan crystals. The plates were then shaken for 15 min on the shaker and optical density of each well was measured at 570 nm on a multi-well ELISA plate (BMG Labtech, Germany) (26). The response (as the growth inhibition rate) was calculated by the following equation:

Inhibitory concentration values (IC_50_) were directly determined by nonlinear regression analysis with GraphPad Prism (27).

*Optimization of the antimicrobial agent production*

All the production experiments were carried out in 250 ml flasks containing 100 ml of the production medium prepared with different nutrients concentration according to the selected factorial design using the same procedure mentioned earlier. The crude bioactive compound was extracted from the broth culture after the removal of mycelia biomass (with centrifugation at 16000×g for 10 min) and then the antibacterial activity of each supernatant was determined (12).

*Anti-MRSA activity determination*

The cell-free supernatant was assayed for its antibacterial activity against *Staphylococcus aureus subsp. aureus *(ATCC^®^ 33591™) (28) (www.atcc.org) in triplicates using the disc diffusion method (29). Briefly, an overnight culture of the MRSA was diluted using Muller-Hinton to an OD_600_ of 0.130, one swab spread on a Muller-Hinton agar plate. The two-fold serial dilutions of each sample were prepared and then 30 µl of each diluted sample was loaded onto 6 mm sterile discs. After drying, the impregnated discs were placed on Mueller-Hinton agar (HiMedia, India) plates which had been previously inoculated with the MRSA inoculum. Sterile discs impregnated with culture broth and sea salt were used as controls. After 24 hr incubating at 37 ^°^C, the diameter of translucent inhibition zones around the discs was measured and plotted on a dose-response curve. The antibacterial activity was calculated using the area under the curve (AUC) (30). 

*Screening experiments using Plackett-Burman design*

PBD was used to find the most significant factors for the production of the antibacterial compound produced by *Streptomyces *sp. MN41. Design-Expert® version 7 was used as the biostatistical software for both experiment and analysis of the data. Nine independent variables were screened by representing them at two levels, low (−) and high (+), using 12 trials (Table 1). The eleven variables assigned in Plackett-Burman designs were A (starch), B (yeast extract), C (peptone), D (KBr), E (Fe_2_(SO_4_)_3_·4H_2_O), F (calcium carbonate), G (sea salt), H (pH), J (agitation), with two “dummy” variables K and L at high and low levels. The experiments were conducted in triplicates and the average antibacterial activity (AUC) against MRSA was noted as the response. Based on the analysis of variance (ANOVA) and Pareto results, the variables with a significant effect on the antimicrobial compound production were determined and then used for further optimization (31). 

*Response surface methodology using central composite design (CCD)*

Optimum levels of four most significant components (Starch, pH, CaCO_3_, and peptone) obtained from the previous Plackett-Burman study were investigated using the CCD function, of the RSM, using Design Expert 7 trial package (Stat-Ease, Inc. Minneapolis, USA). The selected significant factors and their levels used in the CCD experiment are given in Table 2. According to the CCD (Table 2) for 4 variables, a total of 30 experiments were carried out simultaneously with five replicates of the central point. The statistical adequacy of the model was determined by using. Overall model significance and quality of the polynomial model equation was judged statistically through the coefficient of determination (R) and adjusted R. Three dimensional response surface plots were drawn to illustrate the relationship between the responses and the experimental levels of each independent variable (30). 

*Experimental validation of the optimization*

The statistical model and the optimization were experimentally validated using both antimicrobial assay and HPLC-derived profile via culturing the strain MN41 under non-optimized and optimized levels of variables (12). The antibacterial activity of the cell-free supernatant was determined as above and also the production profile under optimized conditions was compared to the non-optimized one using HPLC analysis performed. 

## Results

***Culture characteristics***

**Figure 1:**
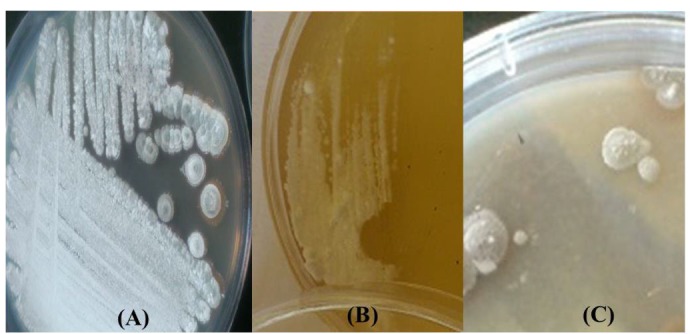
Colony morphology of *Streptomyces* sp. MN41 using some culture media (A: SCA , B: ISP2, C: ISP3)

The *Streptomyces *sp. MN41 developed a truly-expand aerial mycelium with suitable sporulation when cultured on different SCA medium. The growth and colony morphology of the tested strain on different media are shown in Table 3 and Figure 1. 

***Phylogenetic characterization***

**Figure 2:**
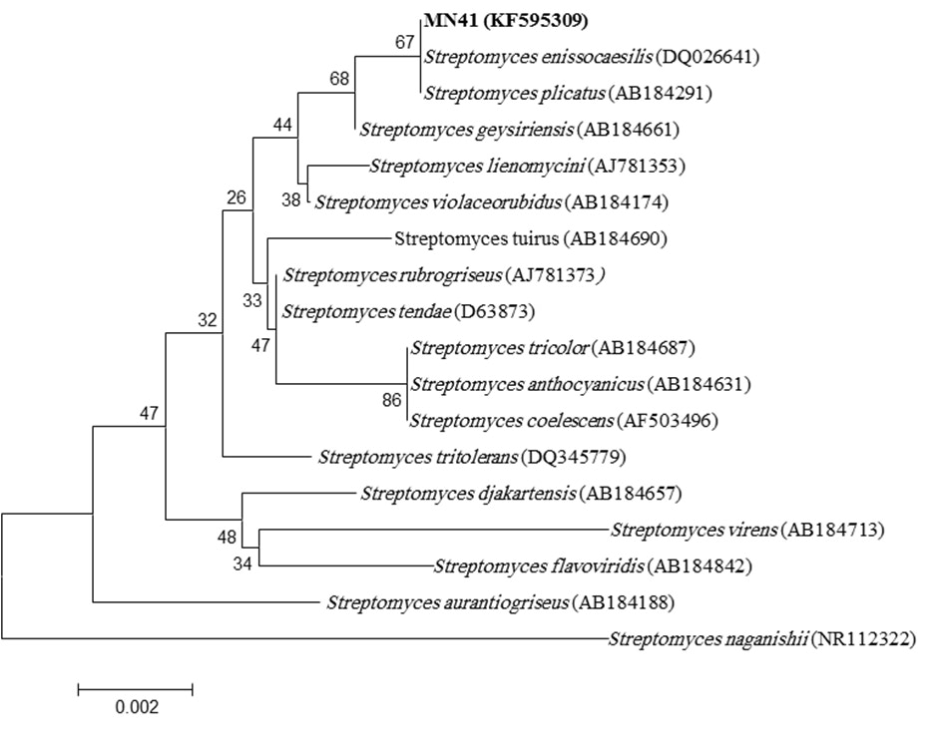
The phylogenetic tree of *Streptomyces* sp. MN41 using 16S rRNA analysis

Using 16S rRNA gene sequence, the phylogenetic tree (Figure 2) of Strain MN41 was created and presented to GenBank with an accession number of KF595309. Strain MN41 was posed in the same branch with *Streptomyces enissocaesilis* (DQ026641) and *Streptomyces plicatus* (AB184291), while *S. geysiriensis* (AB184661) was observed to be the near strain. Strain MN41 was put with *S. enissocaesilis* (DQ026641) on the same branch with 99.7% sequence homology.

***Identification analysis***

**Figure 3:**
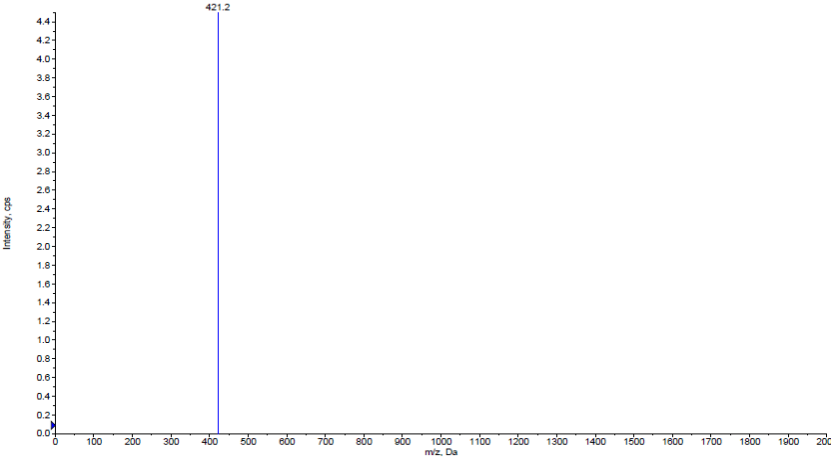
Mass spectrum of the antibacterial compound isolated from *Streptomyces* sp. MN41

After fractionation of organic solvent-extract via using silica gel column,, the active fractions were evaluated on TLC followed by a semi-preparative HPLC for the fractions (Fr 82-89) that exhibited a single band on TLC analysis. Using the semi-preparative HPLC, the sub-fraction that displayed antibacterial activity was coming out after about 5.8 min. The molecular weight of bioactive agent was observed to be 421.2 using Mass spectrometry. (Figure 3).

**Figure 4:**
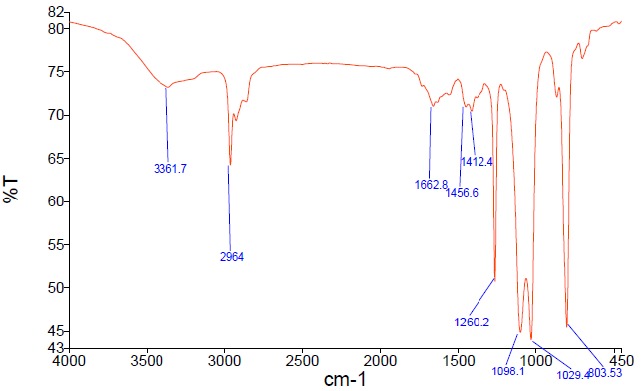
Fourier-transform infrared spectroscopy of the bioactive compound produced by *Streptomyces* sp. MN41

Hydrogen NMR (^1^H-NMR) was performed to elucidate the probable group in the structure of the purified bioactive compound followed by carbon NMR (^13^C-NMR) and 2-dimensional NMR. Table 4 describes the ^1^H-NMR and ^13^C-NMR spectrums of the active compound. ^1^H-NMR peaks at 6.72 ppm, 6.81 ppm and 6.06 revealed the presence of the aromatic group, probably pyrrole ring. Although pyrrole ring contains four H, it seems here there is a bond at α position with external group and for other three H there are *dd* J-values of β carbon (3.7 & 1.47), β′ carbon (3.7 & 2.55) and α′ carbon (2.47 & 1.5), which was confirmed by ^13^C-NMR peaks at 109 ppm, 114.5 ppm and 122.3 ppm which are belonged to carbon atoms in a pyrrole ring. Position 123.7 with low intensity belongs to a bonded carbon which does not have any free H and explains its low intensity. 2-dimensional NMR results (data not shown) depicts the interaction of these three ^1^H-peaks to each other and to the related ^13^C-peaks, in which it verified the presence of a pyrrole group. A broad stretching peak at 3361.7 cm^−1^ of FTIR spectrum with a peak at 1662.8 cm^-1^ elucidate the presence of –NH and carbon double bond, respectively, which verify the presence of a pyrrole group (Figure 4). As a result, the overall spectral analysis indicated the presence of a natural pyrrole-derivative.

***Cytotoxic activity of active compound using MTT assay***

*In vitro* cytotoxicity assays of the active compound against Hela and Vero cell lines revealed that the active compound has exhibited considerable anti-proliferative activities. The IC_50_ value of the natural pyrrole-derivative compound was calculated at 14.62 µg/ml against Hela cell lines, while 59.37 µg/ml as an IC_50_ was obtained when Vero cell lines used in MTT assay.

***MIC and MBC***

 The active compound showed antibacterial effects against MRSA strain and its MIC against MRSA was measured at 2.80 µg/ml while the MBC was 5.62 µg/ml.

***Screening for essential components on the production of active compound using PBD***

**Figure 5:**
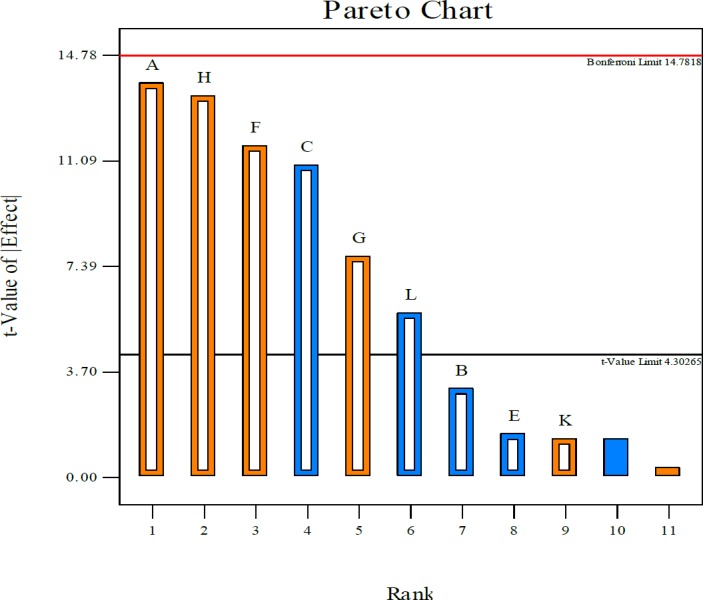
Pareto chart demonstrates how different factors affect the production of the active compound

Estimated effects of variables in the antibacterial compound production from PB-designed experiments are shown in Table 5. Pareto chart (Figure 5) reveals that the mainvariables influencing the production of antimicrobial agent were Starch, pH, CaCO_3_, and peptone. Table 6 represents the Sum of Squares, standard error, *P-value* and Coefficient Estimate of each component from the result of antimicrobial assay given in Table 6.

***Optimization of the selected variables***

The optimum levels of the significant variables including starch, pH, CaCO_3_, and peptone in the production of the antibacterial compound produced by *Streptomyces *sp. MN41 were further optimized using the RSM based on CCD. A total of 30 separate experiments were performed, each in triplicate. The coded levels of the independent variables are given in Table 5. Multiple regression analysis was applied to the experimental data by the CCD design. The Results were fitted with a second-order full polynomial equation. The empirical relationship between antibacterial metabolite production and the 4 test variables obtained from the application of RSM is given by below equation:

The experimental antimicrobial activity and Predicted Response along with CCD has been showed in Table 7. Design Expert software analyzed the regression where Y-axis represents the zone of inhibition (AUC) as a scale of antibacterial activity and the amount of starch, pH, CaCO_3_ and peptone were analyzed using A, B, C and D axes, respectively. Table 8 shows the results of ANOVA analysis of the model. From the F-value of 2.53 and a low* P-value* of 0.0429 it could be dedicated that the model is extremely significant. In addition, the experimental data observed were in a proper fit as the lack of fit of the model with a value of 3.77 was insignificant. “Adeq Precision” measures the signal to noise ratio. A ratio greater than 4 is desirable. The ratio of 5.208 indicates an adequate signal. The coefficient of variation of the model was 18.93% and the PRESS statistic value of 269.85 proved the validity of the model.

**Figure 6:**
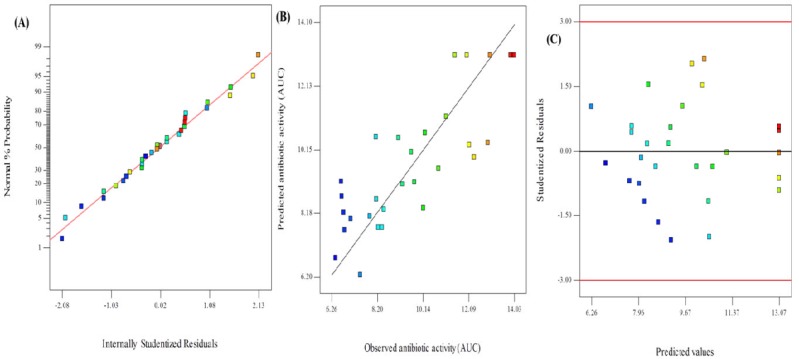
Diagnostic plots showing the model adequacy. (A) normal probability plot of the studentized residuals (B) Plot of observed response vs predicted response and. (C) Internally studentized residuals versus predicted response plot

To evaluate the model competence and determine the signs of problems in the data obtained, diagnostic plots were adopted. A linear pattern in the normal probability plot which is depicted in Figure 6A disproves the presence of any problem in the normality of the data. Figure 6B indicates the acceptable accordance of obtained-antimicrobial response versus predicted response. Figure 6C represents a plot of studentized residuals versus predicted values which has been used for evaluating the presence of constant errors. Residuals displayed randomness in scattering and propose that the variance of observation was constant. 

**Figure 7:**
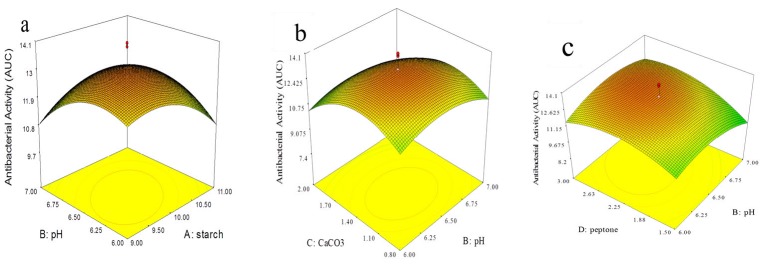
Response surface 3D plots showing individual and interactive effects of variables on antibacterial compound production produced by *Streptomyces* sp. MN41. (a) Effects of starch and pH on antibacterial activity. (b) Effects of CaCO_3_ and pH on antibacterial activity. (c) Effects of pH and peptone on antibacterial activity

The interactions between the two factors and their optimum levels were evaluated using the response surface 3D plots (Figure 7), Figure 7a depicts how Starch, and pH influence the production of the antibacterial compound. Using a moderate concentration of starch, the antibacterial activity will be raised by decreasing the pH. The same trend was observed when the interactive effect of CaCO_3_ with pH (Figure 7b) on the production of the antimicrobial agent was evaluated, while a high and low level of pH resulted in a negative effect when interacts with peptone (Figure 7c).

The maximum antimicrobial activity was predicted to be 13.07 AUC, when the values of variables were as starch = 10 mg/l, CaCO_3_ = 1.4 g/l, peptone 2.25 g/l and pH = 6.5.

***Experimental validation***

Both antibacterial activity determination and HPLC profile analysis were used to validate the optimized amount of variables predicted by RSM. There is an accordance between the average antibacterial activity obtained experimentally and the predicted one wich were 13.87 AUC and 13.07 AUC, respectively (Table 9). Consequently, the accuracy and reliability of the model to predict the bioactive metabolite production produced by strain MN41 is scientifically admissible. 

**Figure 8:**
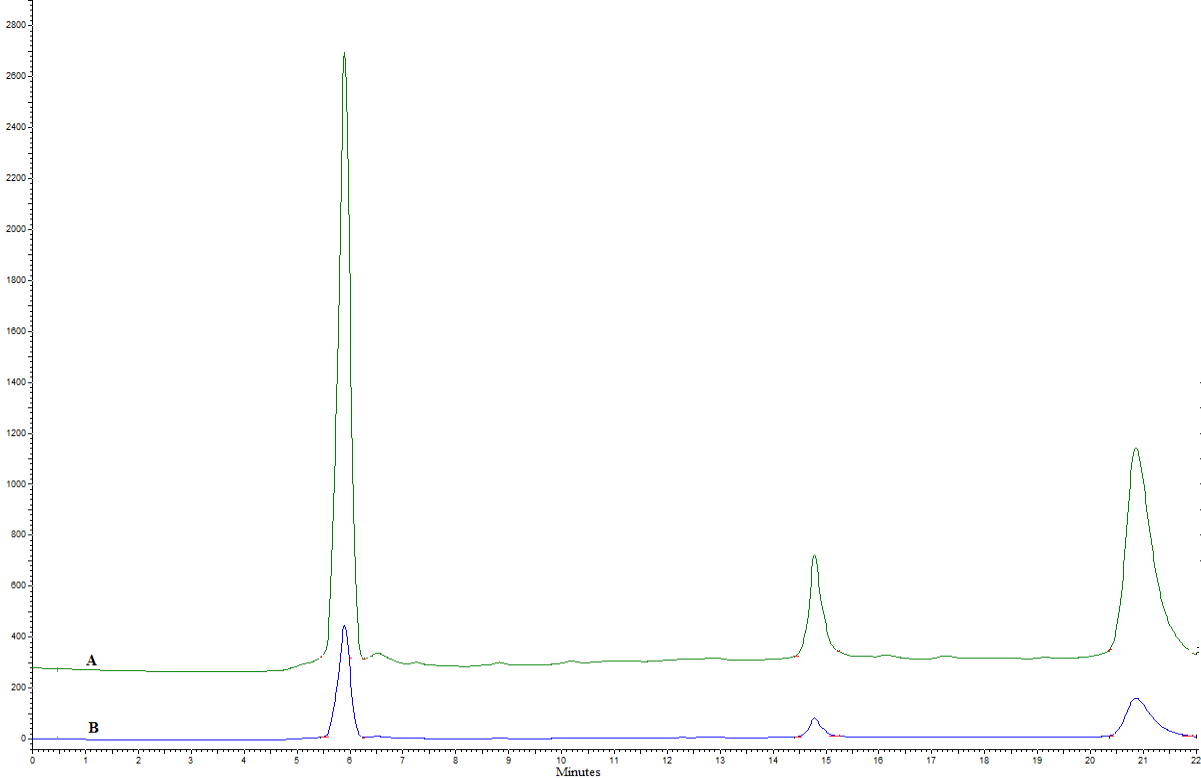
HPLC analysis of bioactive compound presented in the cell-free supernatant of strain MN41 cultivated in (A) optimized and (B) non-optimized amount of culture conditions

HPLC analysis of the cell-free supernatant under non-optimized and optimized amount of variables indicated that using the latter conditions substantially increased the bioactive compound production (Figure 8).

These findings affirmed that the optimization of the production of active biomolecule was forcefully favored using biostatical model.

## Discussion

Over the past decades, the marine ecosystem has gained progressive interest as a unique under-studied source of new value-added compounds in particular bio-active compounds, by both the industry and academia. Many marine bacteria, especially *Streptomyces* isolated from marine habitats have been reported as potential producers of novel secondary metabolites with antibiotic properties (7, 8, 32). Regarding the real demand of new antibacterial agents to fight against antimicrobial-resistant microorganisms treat, this investigation was tried to characterize a marine *Streptomyces* known to produce an antibacterial compound, and optimize its culture conditions to improve the antimicrobial molecules production (12, 33). 

Using the widely-used cultural characterization methods for identification of actinomycetes, the isolated bacterium MN41 was identified as *Streptomyces* species molecular based methods (34). In addition, the phylogenetic analysis emerged from 16S rRNA gene indicated that MN41 is a neighbouring strain of *Streptomyces enissocaesilis* (DQ026641) (35) with 99.7% sequence similarity. Since MN41 tolerates sodium chloride concentration up to 40 g/l, but grows optimally in a mild NaCl concentration which reveal this strain should be a moderately halo-tolerant *Streptomyces*. More than 124 compounds produced by *Streptomyces* had been reported to have moderate to potent anti- MRSA activity, for example, polyketomycin, heliquinomycin, griseusin A, citreamicin A, chaxamycin D, nosiheptide and marinopyrrole A which show lower MIC than several antibiotics such as vancomycin (36).

**Table 1:** Range and levels of different process variables used in Plackett-Burman design for antibacterial activity

**Medium components**	**Codes**	**High values (+)**	**Low values(–)**
Starch	A	14 g/l	7 g/l
Yeast extract	B	6 g/l	3 g/l
Peptone	C	3 g/l	1 g/l
KBr	D	0.2 g/l	0.05 g/l
Fe_2_(SO_4_)_3_.4H_2_O	E	0.06 g/l	0.02 g/l
CaCO_3_	F	3 g/l	0.5 g/l
Sea salt	G	40 g/l	10 g/l
pH	H	8	6
Agitation	J	200 rpm	150 rpm

**Table 2:** Range and levels of different process variables used in Central composite design for antibacterial activity

**Medium components**	**Codes**	**+**	**0**	**–**
Starch	A	12 g/l	11 g/l	10 g/l
pH	B	7	6	6
CaCO_3_	C	2 g/l	1.4 g/l	0.8 g/l
Peptone	D	3.5 g/l	2.25 g/l	1.5 g/l

**Table 3:** Cultural characteristics of strain MN41 on different media

**Medium**	**Growth**	**Aerial mycelium**	**Substrate mycelium**
Starch casein agar (SCA)	Good	Brown- white	Brown
Extract malt extract agar (ISP2)	Good	Creamy white	yellow
Oat meal agar (ISP3)	Good	Brown- white	Dark brown
Inorganic salt starch agar (ISP4)	Good	Brown	yellow
Glycerol asparagine agar (ISP5)	Good	White	Brown
Peptone yeast extract agar (ISP6)	Good	Brown	Mint cream
Tyrosine agar (ISP7)	Good	Brown	Brown- white

**Table 4:** Hydrogen NMR and carbon NMR spectrum of the bioactive compound

	Position (ppm)	Probable group	J value
^1^H-NMR	**6.06**	**Aromatic group (pyrrole ring)**	**dd (3.7 & 2.55)**
	**6.72**	**Aromatic group (pyrrole ring)**	**dd (3.7 & 1.47)**
	**6.81**	**Aromatic group (pyrrole ring)**	**dd (2.47 & 1.5)**
^13^C-NMR	**109**	**Aromatic carbon**	
	**114.5**	**Aromatic carbon**	
	**122.3**	**Aromatic carbon**	
	**123.7 (low int.)**	**Aromatic carbon (with no free H)**	

**Table 5:** Statistical analysis of effects of variables on antibacterial activity in two levels (L and H) using Plackett-Burman design design

Run	Variables											Antibacterial activity against MRSA (AUC)
	A	B	C	D	E	F	G	H	J	K	L	
1	H	H	H	L	H	H	H	L	L	L	H	3.035
2	H	L	H	H	L	H	H	H	L	L	L	14.073
3	H	L	L	L	H	L	H	H	L	H	H	12.045
4	H	L	H	H	H	H	L	L	H	L	H	2.032
5	H	H	H	L	L	H	H	H	H	H	L	5.795
6	L	L	H	L	H	H	L	H	H	H	L	7.250
7	H	H	L	H	H	H	L	L	L	H	L	10.158
8	L	H	L	H	L	H	H	H	H	L	L	8.102
9	L	L	L	L	L	L	L	L	L	L	L	3.612
10	L	L	L	H	L	H	L	L	H	H	H	7.676
11	H	H	L	L	L	H	H	L	H	L	H	11.913
12	L	H	H	H	L	L	L	H	L	H	H	2.232

**Table 6:** Statistical analyses of effects of variables on antibacterial activity as per Plackett-Burman design

**Variables**	**Codes**	**Standard error**	**Sum of squares**	**df**	***P-value***	**F** **value**
Starch	A	0.15	48.43	1	0.0052	190.94
Yeast extract	B	0.15	2.48	1	0.0889	9.77
Peptone	C	0.15	30.37	1	0.0082	119.73
Fe_2_(SO4)_3_.4H_2_O	E	0.15	0.60	1	0.2643	2.36
CaCO_3_	F	0.15	34.30	1	0.0073	135.22
Sea salt	G	0.15	15.25	1	0.0162	60.14
pH	H	0.15	45.27	1	0.0056	178.48
KBr	D	0.15	0.48	1	0.3045	1.87
Agitation	J	0.15	8.43	1	0.0288	33.23

**Table 7:** Central composite design matrix in coded values and responses

Run	Variables				Experimental response	**Predicted** response
	Starch (g/l)	pH	CaCO_3_ (g/l)	Peptone (g/l)		
**1**	**11.00**	**6.00**	**0.80**	**3.00**	**6.671**	**9.16**
**2**	**9.00**	**6.00**	**0.80**	**1.50**	**8.147**	**10.54**
**3**	**9.00**	**6.00**	**2.00**	**3.00**	**6.6955**	**8.69**
**4**	**10.00**	**6.50**	**1.40**	**3.75**	**12.905**	**10.36**
**5**	**11.00**	**7.00**	**0.80**	**3.00**	**10.2345**	**10.67**
**6**	**10.00**	**6.50**	**1.40**	**0.75**	**8.241**	**7.72**
**7**	**9.00**	**6.00**	**0.80**	**3.00**	**11.135**	**11.18**
**8**	**10.00**	**6.50**	**1.40**	**2.25**	**14.012**	**13.07**
**9**	**11.00**	**6.00**	**2.00**	**3.00**	**8.1655**	**8.60**
**10**	**9.00**	**7.00**	**0.80**	**1.50**	**8.485**	**8.29**
**11**	**10.00**	**7.50**	**1.40**	**2.25**	**9.786**	**9.14**
**12**	**10.00**	**6.50**	**1.40**	**2.25**	**13.88**	**13.07**
**13**	**10.00**	**5.50**	**1.40**	**2.25**	**12.325**	**9.92**
**14**	**11.00**	**7.00**	**0.80**	**1.50**	**6.8105**	**7.65**
**15**	**8.00**	**6.50**	**1.40**	**2.25**	**12.115**	**10.30**
**16**	**12.00**	**6.50**	**1.40**	**2.25**	**10.81**	**9.57**
**17**	**10.00**	**6.50**	**2.60**	**2.25**	**7.484**	**6.26**
**18**	**10.00**	**6.50**	**1.40**	**2.25**	**14.03**	**13.07**
**19**	**11.00**	**7.00**	**2.00**	**3.00**	**9.645**	**10.08**
**20**	**11.00**	**7.00**	**2.00**	**1.50**	**7.883**	**8.07**
**21**	**11.00**	**6.00**	**2.00**	**1.50**	**6.7735**	**8.19**
**22**	**9.00**	**7.00**	**2.00**	**3.00**	**7.0745**	**7.07**
**23**	**9.00**	**7.00**	**0.80**	**3.00**	**9.106**	**10.52**
**24**	**9.00**	**7.00**	**2.00**	**1.50**	**6.4345**	**6.78**
**25**	**10.00**	**6.50**	**1.40**	**2.25**	**11.511**	**13.07**
**26**	**9.00**	**6.00**	**2.00**	**1.50**	**9.2755**	**9.07**
**27**	**10.00**	**6.50**	**1.40**	**2.25**	**13.002**	**13.07**
**28**	**10.00**	**6.50**	**1.40**	**2.25**	**12.012**	**13.07**
**29**	**11.00**	**6.00**	**0.80**	**1.50**	**8.4105**	**7.73**
**30**	**10.00**	**6.50**	**0.20**	**2.25**	**10.1625**	**9.33**

**Table 8:** Summary of ANOVA for response surface quadratic model using Central composite design

**Source**	**Sum of squares**	**df**	**Mean square**	**F Value**	***p-value ***	**Significance**
Model	121.06	14	8.65	2.53	0.0429	significant
Residual	51.36	15	3.42	-	-	-
Lack of fit	45.34	10	4.53	3.77	0.0781	Not significant
Pure error	6.02	5	1.20	-	-	-
Core total	172.43	29	-	-	-	-

**Table 9:** The production of antibacterial agent produced by strain MN41 under non-optimized and optimized amount of variable

**Culture conditions**	**Level of variables**				**Antibacterial activity ** **(AUC)**
	Starch (g/l)	pH	CaCo_3 _(g/l)	Peptone (g/l)	
Non-optimized	9	7	2	1.5	6.34
Optimized (predicted)	10	6.5	1.4	2.25	13.07
Optimized (experimental)	10	6.5	1.4	2.25	13.87

We isolated a pyrrole-like bioactive compound from *Streptomyces *sp. MN41 with a desirable anti-MRSA activity, in which its MIC and MBC were measured at 2.80 µg/ml and 5.62 µg/ml, respectively. Its antibacterial effect against MRSA is comparable to the previously reported antimicrobials with a pyrrole-like structure: Moenomycin A (37), nosokomycins A- D and angumicynone B (38) which also were isolated from marine *Streptomyces* and reported as potential marine drugs against MRSA. The observed anti-MRSA MIC of the pyrrole-derivative compound from *Streptomyces* sp. MN41 (2.8 μg/ml) was lower than that of angumicynone B (12.5 μg/ml) and moenomycin A (4 μg/ml), and higher than nosokomycins A- D 0.125 μg/ml (36). Moreover, this MIC is lower than vancomycin (4–8 μg/ml), commercially available antibiotics (39). 

Thus, the observed MIC and MBC of the isolated pyrrole derivative compounds in our study, demonstrate that: I) the compound exhibited a significant antimicrobial activity against MRSA; II) compared to the previous compounds it can be considered as a potential anti-MRSA compound for further study’ and III) it may exhibit effective antibacterial activity against other drug- or multidrug-resistant bacteria especially other *Staphylococcus* and related genus. 

Mass analysis, as a sensitive, rapid and reliable technique for detecting and identifying metabolites (40), showed that MN41 produces an antibacterial agent with a molecular weight of 421.2. ^1^H-NMR, ^13^C-NMR, 2-dimensional NMR determination and IR analyses demonstrated that the compound is a pyrrole-derivative. It has been shown that pyrrole derivatives compounds possess diverse desirable activities as a drug, for example, antimicrobial activity, anti-inflammatory activity (41), and antitumor activity (42). Hughes *et al.* isolated marinopyrroles with anti-MRSA activity from a marine *Streptomyces* sp. The pyrrole-like structure of an antimicrobial not only suggest a desirable antimicrobial effect but also offers chemical flexibility to further development and enhancement of the original structure, which may lead to improvement of the desired activity (43).

Many antibacterial agents also display antitumor activity. The rapid development of multiple drugs resistance cases in tumor chemotherapy has urged searching for novel agents. Our results demonstrated that the pyrrole derivative compound from *Streptomyces* sp. MN41, in addition to the anti-MRSA activity, had antitumor activity against the tested cell lines (with a IC_50_ of 14.62 µg/ml and 59.37 µg/ml against Hela and Vero cell lines, respectively). It is reported that if a compound displays an IC_50_ value less than 30 µg/ml against cancer cell, it is considered as a potential antitumor agent and has the potential for further drug development studies (26, 44). Thus, the observed anticancer effects of the isolated compound in our study suggest its potential application as an antitumor agent, in particular against multi-drug resistant cancer cell lines.

Despite the fact that *Streptomyces sp.* MN41 produced this antibacterial agent, optimization of the production process is vital for a commercially viable production process. PBD and RSM have been used as two effective statistical tools for such task (31). For example, Wang *et al.* (15) applied RSM approach in a medium optimization study for producing an active compound by *Xenorhabdus* *bovienii* and reported a 37.8% increase in production. Rajeswari *et al.* (12) reported a 78.8% increase in production of an antibacterial by *Streptomyces *sp*. *JAJ13 using RSM approach. RSM approach with CCD was used to increase active compound production in several *Streptomyces* species such as *Streptomyces* *sindenensis* (45), *Streptomyces daufpe* 3060 (46) and *Streptomyces alboflavus* 313 (47).

In our study, the results of PBD revealed that the crucial factors affecting the production of the bioactive compound by strain MN41 were starch, pH, CaCO_3_, and peptone. Although with regard to the marine habitat of the microorganism (isolated from marine sediments) (11) it was expected that NaCl salt concentration would be an influencing factor, the PBD models proved us wrong. Raytapadar and Paul (48) reported starch as a significant media component for the production of the antibacterial compound from *Streptomyces aburaviensis* 1DA-28. Similarly, CaCO_3_ affects the production of cyclic hexapeptide antibiotic by *Streptomyces alboflavus* (47). 

Further optimization of the influencing factors using RSM with allowed us to determine the optimum levels of media components. Our results demonstrated that optimization of the condition according to the PDB and RSM model resulted in 217% increase in antibacterial compound production by strain MN41, in which the R2 value was calculated at 0.702, indicating the model can explain 95.0% of the total variations. The confirm of fit of the response surface model is checked using the coefficient of determination (R2), which provides a measure of variability in the observed response explained by the experimental factors and their interactions (12, 15). The nearer R2 value to 1.00, the higher accuracy of the model to predict the responce. Therefore, considering the calculated R2, indicated that the developed experimental design was accurate in optimizing the selected culture conditions. 

For further studies, we suggest x-ray crystallography as a complementary tool for structure determination. In addition, evaluating the LC_50_ value of the purified compound is valuable to determine its cytotoxic effect. 

## Conclusion

*Streptomyces* sp. MN41 produced a bioactive compound with a pyrrole-like structure. This pyrrole derivative showed significant anti-MRSA and antitumor activities. To further enhance the production yield, the process was subjected to PBD and RSM modeling in which starch, pH, CaCO_3_ and peptone were determined as significant factors influencing antibacterial compound production, and optimization of such factors resulted in 218% increase in the production of the anti-MRSA compound. The pyrrole-like structure of the compounds demonstrates a great potential for further drug development, e.g. enhancement of chemical structure, and the observed increase in the production level of the compound by the bacteria after optimizing the culture condition suggest that there is a room for future enhancement by e.g. engineering of the culture condition or the bacterial genome.

## References

[1] Maragakis LL, Perencevich EN, Cosgrove S (2008). Clinical and economic burden of antimicrobial resistance. Expert Rev Anti Infect Ther.

[2] Loomba PS, Taneja J, Mishra B (2010). Methicillin and Vancomycin resistant S aureus in hospitalized patients. J Glob Infect Dis.

[3] Enright MC (2003). The evolution of a resistant pathogen-the case of MRSA. Curr Opin Pharmacol.

[4] Subramani R, Aalbersber W (2012). Marine actinomycetes: an ongoing source of novel bioactive metabolites. Microbiol Res.

[5] Dharmaraj S (2010). Marine Streptomyces as a novel source of bioactive substances. World J Microbiol Biotechnol.

[6] Arasu MV, Duraipandiyan V, Ignacimuthu S (2013). Antibacterial and antifungal activities of polyketide metabolite from marine Streptomyces sp AP-123 and its cytotoxic effect. Chemosphere.

[7] Jang KH, Nam SJ, Locke JB, Kauffman CA, Beatty DS, Paul LA (2013). Anthracimycin, a potent anthrax antibiotic from a marine-derived actinomycete. Angew Chem In Ed Engl.

[8] Jiao W, Zhang F, Zhao X, Hu J, Suh JW (2013). A novel alkaloid from marine-derived Streptomycesxinghaiensis with broad-spectrum antibacterial and cytotoxic activities. PloS one.

[9] De Carvalho CCCR, Fernandes P (2010). Production of metabolites as bacterial responses to the marine environment. Mar Drugs.

[10] Lam KS (2006). Discovery of novel metabolites from marine actinomycetes. Curr Opin Microbiol.

[11] Mohseni M, Norouzi H, Hamedi J, Roohi A (2013). Screening of antibacterial producing actinomycetes from sediments of the Caspian Sea. Int J Mol Cell Med.

[12] Rajeswari P, Jose PA, Amiya R, Jebakumar SRD (2015). Characterization of saltern based Streptomyces sp and statistical media optimization for its improved antibacterial activity. Front Microbiol.

[13] Arul Jose P, Satheeja Santhi V, Jebakumar SR (2011). Phylogenetic-affiliation, antimicrobial potential and PKS gene sequence analysis of moderately halophilic Streptomyces sp inhabiting an Indian saltpan. J Basic Microbiol.

[14] Greasham RL (1983). Media for microbial fermentations.

[15] Wang Y, Fang X, An F, Wang G, Zhang X (2011). Improvement of antibiotic activity of Xenorhabdusbovienii by medium optimization using response surface methodology. Microb Cell Fact.

[16] Kanmani P, Karthik S, Aravind J, Kumaresan K (2012). The use of response surface methodology as a statistical tool for media optimization in lipase production from the dairy effluent isolate Fusarium solani. ISRN Biotechnol.

[17] Jose PA, Jebakumar SRD (2013). Diverse actinomycetes from Indian coastal solar salterns-a resource for antimicrobial screening. J Pure Appl Microbiol.

[18] Shirling EB, Gottlieb D (1966). Methods for characterization of Streptomyces species. Int J Syst Evol Microbiol.

[19] Stephen JR, McCaig AE, Smith Z, Prosser J, Embley TM (1996). Molecular diversity of soil and marine 16S rRNA gene sequences related to beta-subgroup ammonia-oxidizing bacteria. Appl Environ Microbiol.

[20] Tamura K, Peterson D, Peterson N, Stecher G, Nei M, Kuma S (2011). MEGA5: molecular evolutionary genetics analysis using maximum likelihood, evolutionary distance, and maximum parsimony methods. Mol Biol Evol.

[21] Wu Z, Li S, Li J, Chen Y, Saurav K, Zhang Q (10428.). Antibacterial and cytotoxic new napyradiomycins from the marine-derived Streptomyces sp. SCSIO.

[22] Saraswathi K, Sabitha Rani AM, Sindhu S, Arumugam P (2015). Isolation, characterization of bioinspired secondary metabolites producing actinomycetes from marine soil samples. Int J Curr Microbiol Appl Sci.

[23] Ebaba SS, Edrada RA, Lin W, Prokesh P (2008). Methods for isolation, purification and structural elucidation of bioactive secondary metabolites from marine invertebrates. Nat Protoc.

[24] Oniszczuk A, Wójtowicz A, Oniszczuk T, Olech M, Nowak R, Wojtunik K (2015). Extruded corn gruels containing linden flowers: quantitation of phenolic compounds and selected quality characteristics. Open Chem.

[25] Soheili V, Khedmatgozar Oghaz N, Sabeti Z, Fazly Bazzaz BS (2016). The novel effect of cis-2-decenoic acid on biofilm producing Psudomonasaeruginosa. Microbiol Res (Pavia).

[26] Ravikumar S, Fredimoses M, Gnanadesigan M (2012). Anticancer property of sediment actinomycetes against MCF-7 and MDA-MB-231 cell lines. Asian Pac J Trop Biomed.

[27] Zheng Z, Zeng W, Huang Y, Yang Z, Li J, Cai H, Su W (2000). Detection of antitumor and antimicrobial activities in marine organism associated actinomycetes isolated from the Taiwan Strait. FEMS Microbiol lett.

[28] Norouzi H, Danesh A, Mohseni M, Rabbani Khorasgani M (2018). Marine Actinomycetes with probiotic potential and bioactivity against Multidrug-resistant bacteria. Int J Mol Cell Med.

[29] Boyle VJ, Fancher ME, Ross RW (1973). Rapid modified Kirby-Bauer susceptibility test with single, high-concentration antimicrobial disks. Antimicrob Agents Chemother.

[30] Marzban A, Ebrahimipour G, Danesh A (2016). Bioactivity of a novel glycolipid produced by a halophilic Buttiauxella sp and improving submerged fermentation using a response surface method. Molecules.

[31] Jose PA, Sivakala KK, Jebakumar SRD (2013). Formulation and statistical optimization of culture medium for improved production of antimicrobial compound by Streptomyces sp. JAJ06. Int J Microbiol.

[32] Kannan RR, Iniyan AM, Prakash VS (2011). Isolation of a small molecule with anti-MRSA activity from a mangrove symbiont Streptomyces sp PVRK-1 and its biomedical studies in Zebrafish embryos. Asian Pac J Trop Biomed.

[33] Chaston JM, Suen G, Tucker SL, Andersen AW, Bhasin A, Bode E (2011). The entomopathogenic bacterial endosymbionts Xenorhabdus and Photorhabdus: convergent lifestyles from divergent genomes. PloS one.

[34] Williams STM, Goodfellow M, Alderson G, Wellington EMP, Sneath PHA, Sackin MJ (1983). Numerical classification of Streptomyces and related genera. J Gen Microbial.

[35] Mousumi D, Dayanand A (2013). Production and antioxidant attribute of L-glutaminase from Streptomyces enissocaesilis DMQ-2. Int J Latest Res Sci Technol.

[36] Kemung HM, Tan LT, Khan TM, Chan KG, Pusparajah P, Goh BH (2018). Streptomyces as a prominent resource of future Anti-MRSA drugs. Front Microbiol.

[37] Haste NM, Thienphrapa W, Tran DN, Loesgen S, Sun P, Nam SJ (2012). Activity of the thiopeptide antibiotic nosiheptide against contemporary strains of methicillin-resistant Staphylococcus aureus. J Antibiot.

[38] Park HB, Le JK, Lee KR, Kwon HC (2014). Angumycinones, A and B, two new angucyclic quinones from Streptomycessp KMC004 isolated from acidic mine drainage. Tetrahedron Lett.

[39] CLSI Methods for Dilution Antimicrobial Susceptibility Tests for Bacteria That Grow Aerobically,; Tenth Edition: Approved Standard; 2015.M07-A10.Wayne, PA:CLSI.

[40] Li XB, Qiao B, Yuan YJ (2006). Differential analysis of secondary metabolites by LC-MS following strain improvement of Streptomyces lydicus AS 42501. Biotechnol Appl Biochem.

[41] Wilkerson WW, Copeland RA, Covington M, Trzaskos JM (1995). Anti-inflammatory 4,5-diarylpyrroles activity as a function of cyclooxygenase inhibition. J Med Chem.

[42] Lee H, Lee J, Lee S, Shin Y, Jung W, Kim JH (2001). A novel class of highly potent, selective, and non-peptidic inhibitor of ras farnesyl transferase (FTase). Bioorg Med Chem Lett.

[43] Hughes CC, Kauffman CA, Jensen PR, Fenical W (2010). Structures, reactivities, and antibiotic properties of the marinopyrroles A-F. J Org Chem.

[44] Suffness M, Pezzuto JM, Hostettman K (1990). Assay related to cancer drug discovery. Methods in plant biochemistry: assay for bioactivity.

[45] Praveen V, Ripathi D, Tripathi CKM, Bihari V (2008). Nutritional regulation of actinomycin-D production by a new isolate of Streptomycessindenensis using statistical methods. Indian J Exp Biol.

[46] Marques DAV, Cunha MNC, Araújo JM, Lima-Filho JL, Converti A, Pessoa-Jr A (2011). Optimization of clavulanic acid production by Streptomyces daufpe 3060 by response surface methodology. Braz J Microbiol.

[47] Guo Z, Shen L, Ji Z, Wu W (2012). Enhanced production of a novel cyclic hexapeptide antibiotic (NW-G01) by Streptomyces alboflavus 313 using response surface methodology. Int J Mol Sci.

[48] Raytapadar S, Paul AK (2001). Production of an antifungal antibiotic by Streptomyces aburaviensis IDA-28. Microbiol. Res.

